# Is transphyseal intramedullary fixation of the distal radius in pediatric fractures a safe procedure? An MRI study

**DOI:** 10.3389/fsurg.2025.1520712

**Published:** 2025-02-20

**Authors:** Marco Giordano, Michela Florio, Silvia Careri, Marco Cirillo, Angelo Gabriele Aulisa, Fabio Massimo Pezzoli, Francesco Falciglia

**Affiliations:** ^1^ Department of General Surgery, Orthopedic Institute, Bambino Gesù Children’s Hospital (IRCCS), Rome, Italy; ^2^ Department of Diagnostic Imaging, Bambino Gesù Children’s Hospital (IRCCS), Rome, Italy; ^3^ Department of Human Sciences, Society and Health, University of Cassino and Southern Lazio, Cassino, Italy

**Keywords:** pinning, transphyseal pinning, paediatric fractures, growth plate, RMN

## Abstract

**Background:**

Radius and ulna fractures are very common in the pediatric population. Despite the use of pinning through the growth plate, which was proposed in the past and is still being used to treat these fractures, an instrumental validation to define this procedure as safe has not yet been done. Because of this, in the absence of reliable data regarding the passage of fixation devices through the growth plate, most surgical techniques used for treating radius and ulna fractures are based on absolute respect for the growth cartilage. We conducted an MRI pilot study to evaluate the presence of any growth disturbances, bone bridge formation across the physis, or premature closure of the cartilage, to verify the correlation between wire diameter and the percentage of lesions tolerated by the growth plate and to confirm the safety of the trans-physeal pinning procedure. To specifically avoid the wrist fracture healing process near the growth plate as possible bias of the study, we enrolled only patients with mid-shaft forearm fractures.

**Materials and methods:**

We evaluated 26 patients with diaphyseal forearm fractures who underwent intramedullary percutaneous transphyseal fixation of the distal radius with a Kirschner wire. Intramedullary K-wire and plaster cast were removed, without a second surgery or anesthesia, about 35-40 days after surgery. A clinical and radiographic evaluation was performed at 1, 3, 6 and 12 months from surgery. We conducted a comparative MRI evaluation of both wrists 12 months after the removal of the K-wire to exclude any growth plate damage related to the passage of the wire through it.

**Results:**

clinical data underlined excellent results in most patients. Radiographic healing was achieved in all cases at three months. No significative cartilage disturbances related to the procedure were found in any patient. An asymmetrical bridge that did not correspond to the wire position was found in some older patients, probably related to the initial phase of the growth plate closure process.

**Conclusion:**

This study demonstrates that the percutaneous trans-physeal technique could become a valid alternative to the standard method, offering a rapid learning curve, shorter surgical times, and reduced healthcare costs.

## Introduction

Fractures of the arm are frequent in skeletally immature children (1, 2). During skeletal development, bones are less stable but much more elastic than in adulthood. These properties explain the higher incidence and the more rapid healing of fractures in children and adolescents. The use of Kirschner wires is widely used for the treatment of wrist and forearm fractures. Still, in the absence of reliable data regarding the passage of fixation devices through the growth plate, most surgical techniques used for treating radius and ulna fractures are based on absolute respect for the growth cartilage. To ensure a correct interpretation of the data and to specifically avoid the potential bias related to the wrist fracture healing process near the growth plate, we enrolled only patients with mid-shaft forearm fractures. These lesions are one of the most common injuries in children, being between 3% and 6% of all pediatric fractures (3, 4). Several studies have shown that this percentage has constantly increased in the last decade because of sports/spare time activities with high risk of trauma, like the widespread use of trampolines or, in younger children, inflatable games (5–7). Treating these injuries is currently controversial, and management protocols are constantly evolving. Closed reduction and cast immobilization remain the most common treatment for the majority of these fractures, especially in younger children. The operative treatment of pediatric forearm fractures should be reserved for cases in which an acceptable alignment with closed manipulation is not possible, or in the presence of neurovascular injury, open fractures, or loss of alignment in follow-up. Internal fixation as a treatment for children's forearm fractures has recently increased, and intramedullary fixation is currently the most commonly preferred technique (8, 9). Surgical treatment must respect the features of the juvenile skeleton. Although pinning across the growth plate is commonly used to treat some fractures in children, orthopedic surgeons generally try to avoid violating the physis. In this regard, the surgical technique used as the gold standard in treating diaphyseal forearm fractures in pediatric age is elastic intramedullary nails (Elastic Stable Intramedullary Nailing – ESIN) (10, 11).

Some studies showed, with a radiographic follow-up, that transphyseal pinning was not associated with early or late complications affecting the growth cartilage (12–15).

Magnetic resonance provides a non-invasive and excellent visualization of structures and the anatomical changes of the growth plate and adjacent joint in multiple planes. Jaramillo et al., along with other authors, described the utility of MRI in evaluating physeal injuries and reported that MRI could help detect minor disruptions of the growth cartilage and show early cartilaginous and vascular changes that lead to growth disturbances (16, 17).

This study aims to evaluate, through post-operative MRI, the presence of physeal disturbance in skeletally immature patients following trans-physeal wire fixation for forearm fractures, and correlate presence of damage with wire diameter and percentage of the growth plate affected by wire passage.

## Materials and methods

### Study design

This is a retrospective monocentric study involving 26 patients (both male and female) with diaphyseal forearm fractures who underwent intramedullary fixation of the radius with a percutaneous transphyseal Kirschner wire at our hospital (Bambino Gesù Children's Hospital – Rome). We conducted a comparative MRI evaluation of both wrists 12 months after the removal of the K-wire to assess the physis.

**Figure 1:**
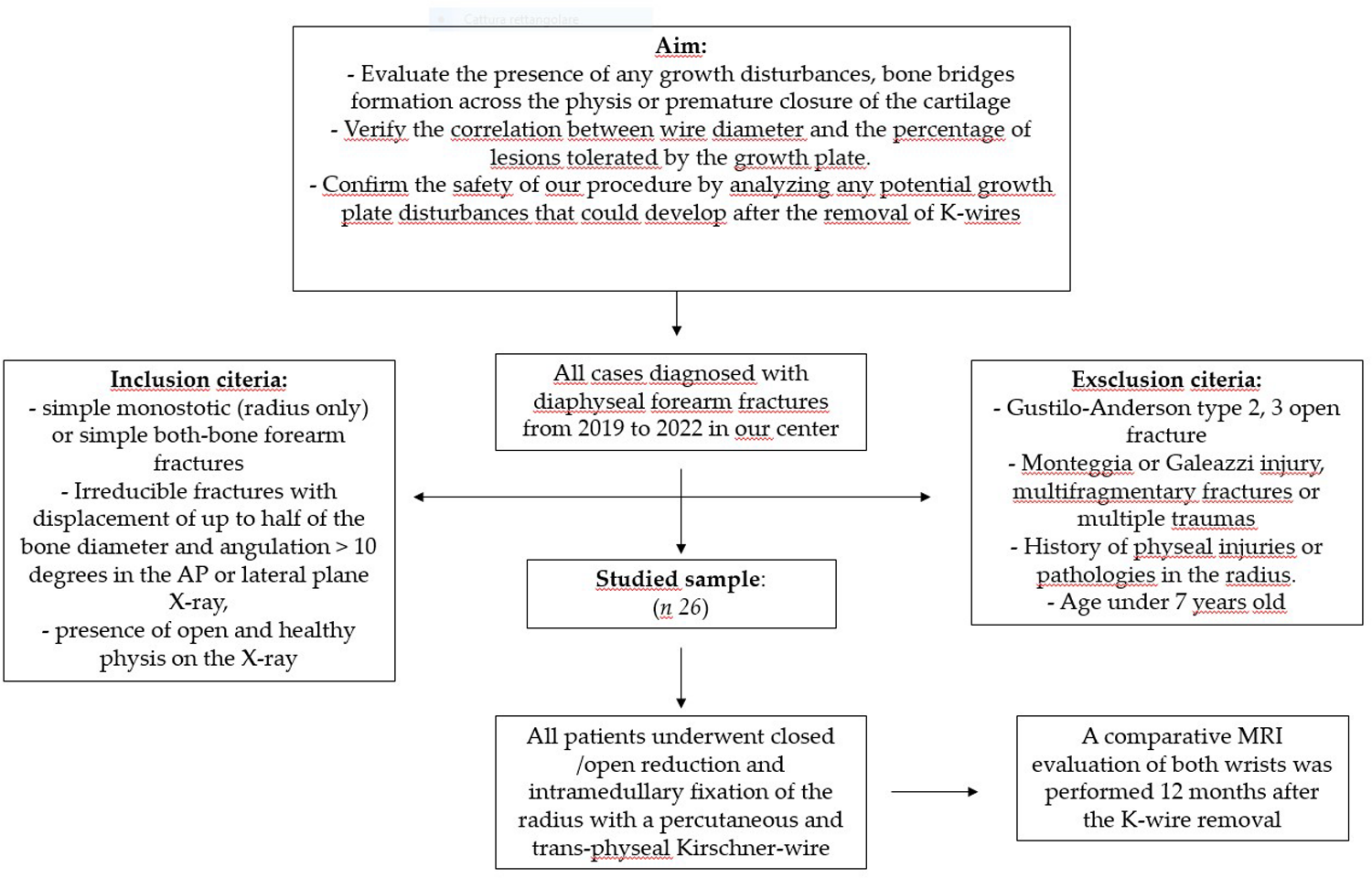
Flow chart for the studied sample selection.

From 2019 to 2022, the patients were prospectively included in this study and were entered into a retrospective database. The mean age was 9.85 years (8–13 years) (Figure 1). Inclusion criteria comprised isolated simple fractures of the radius (22r-D/4.1,22r-D/5.1), both-bone simple forearm fractures (22-D/4.1, 22-D/5.1), according to the AO (Arbeitsgemeinschaft für Osteosynthesefragen) Pediatric Comprehensive Classification of Long Bone Fractures (18, 19), irreducible fractures with displacement of up to half of the bone diameter and angulation >10 degrees in the AP or lateral plane x-ray, presence of open and healthy physis on the x-ray. Patients were excluded from the study if they had Gustilo-Anderson type 2 or 3 open fractures, Monteggia or Galeazzi injuries, multi fragmentary fractures, or multiple traumas, a history of physeal injuries or pathologies in the radius, or were under the age of 7 (as they cannot undergo an MRI examination without sedation). Patients underwent closed or open fracture reduction and intramedullary fixation of the radius with a percutaneous and trans-physeal Kirschner-wire (2–2.5 mm in diameter, according to the patient's age and bone size). After surgery, a long plaster cast was applied before waking up from anesthesia. Intramedullary K-wire and plaster cast were removed, without a second surgery or anesthesia, about 35–40 days after surgery (time of expected consolidation of the fractures, according to patient's age), and a new short plaster cast was applied for the other 20–25 days. Then, a program of daily exercises, usually performed at home, was followed until complete elbow, wrist, and forearm range of motion (ROM) recovery. A clinical evaluation was performed at 1, 3, 6, and 12 months from surgery. Wrist and elbow flexion/extension and forearm pronation/supination ROM were assessed. The uninjured arm was evaluated first, and the affected arm was evaluated about 2–3 min later. Clinical results have been elaborated through Daruwalla's scoring system (20). Angulation and rotation of the fragments were measured on AP and lateral x-rays at presentation, post-surgery, 1, 3 and 12 months from surgery. Furthermore, we evaluated radial and ulnar residual malalignment and radial and ulnar residual displacement, forearm overgrowth, premature radial physeal closure or growth disturbance, and radio-ulnar length discrepancy at the healing time. All measurements were made twice by the same operator, using Carestream digital radiographs. Translation and rotation were evaluated according to International Guidelines (21). Sport restrictions ranged from 4 to 6 months after surgery. The average consolidation time (time elapsed from the osteosynthesis to the first radiographic evidence of bone healing) was calculated.

**Figure 2:**
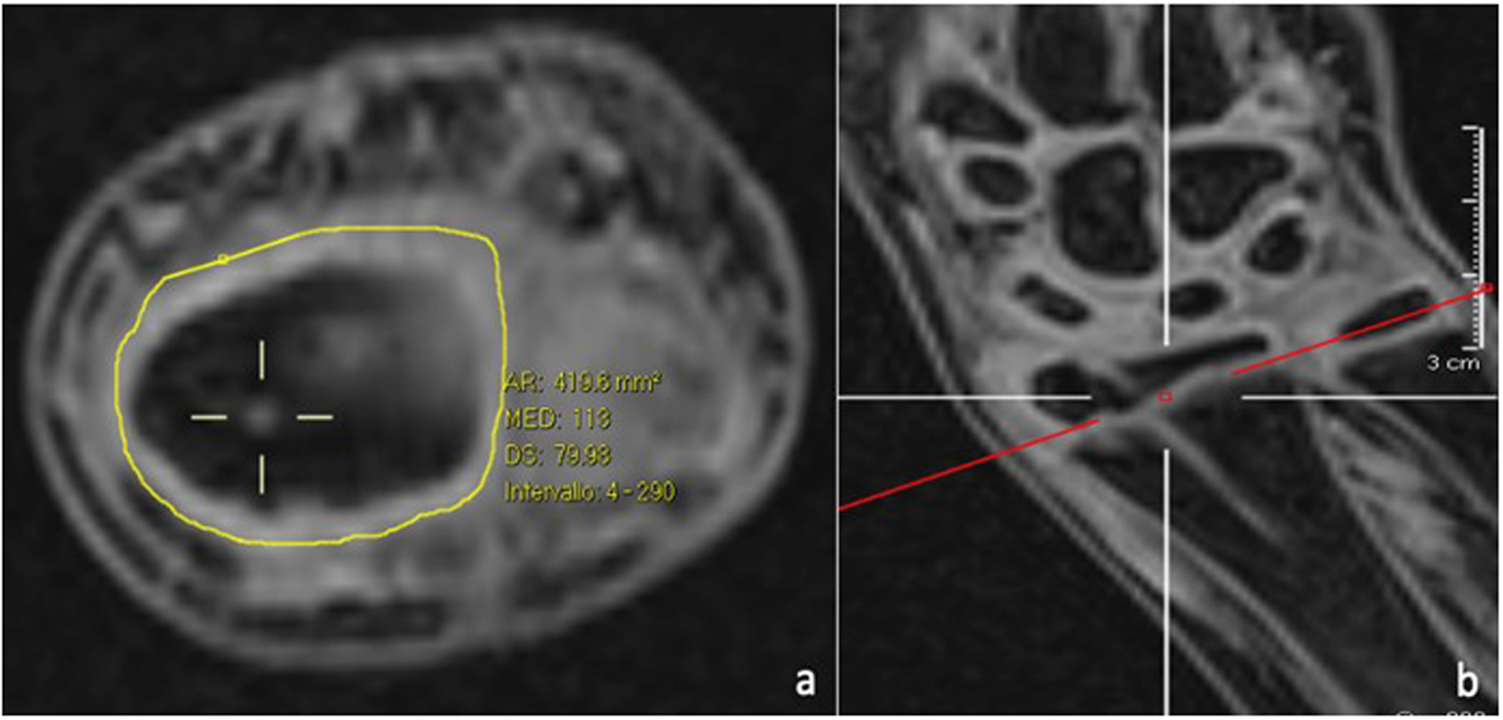
The transphyseal lesion after K-wire removal, as seen on MRI axial **(a)** and coronal **(b)** views, is estimated as a percentage of the total surface are of the physis.

A comparative MRI evaluation of both wrists was performed 12 months after the K-wire removal to:

− evaluate the presence of any growth disturbances, bone bridge formation across the physis, or premature closure of the cartilage;
− verify the correlation between wire diameter and the percentage of lesions tolerated by the growth plate (Figure 2);
− confirm the safety of the trans-physeal pinning procedure.

### MRI imaging tecnique

All patients were examined using a 1,5 Tesla scanner (Siemens MAGNETOM Sola -Siemens Healthcare, Erlangen, Germany-). Participants were placed in the prone position, head first. A 4-channel flex coil was placed, covering both wrists. The exam protocol was based on 3D-sequences-isotropic sequences obtained with a simultaneous coronal scan of both wrists. Field of view and matrix resolution were set to get a voxel size <1 mm in all patients. These features allow high-quality reformats to yield multiplanar images from the original dataset; this is essential to perform an accurate quantitative analysis of the physis because its shape, smooth and flat in infants, becomes oblique and undulated with age. The exam protocol included a 3D Volumetric Interpolated Breath-hold Examination (VIBE), 3D Proton density (DP), and 3D T2 SPACE Short Tau Inversion Recovery (STIR), with a total exam time between 10 and 15 min.

The normal cartilagineous signal of the growth plate was identified in 3D VIBE sequences as a bright modulated line in contrast with the dark bone signal of ossified epiphysis or metaphysis. Physiological complete or incomplete bridges related to normal skeletal maturation were visualized as hypointense areas in all sequences, especially in 3D VIBE, in the absence of anomalies of epiphysis or metaphysis.

### Surgical technique

**Figure 3:**
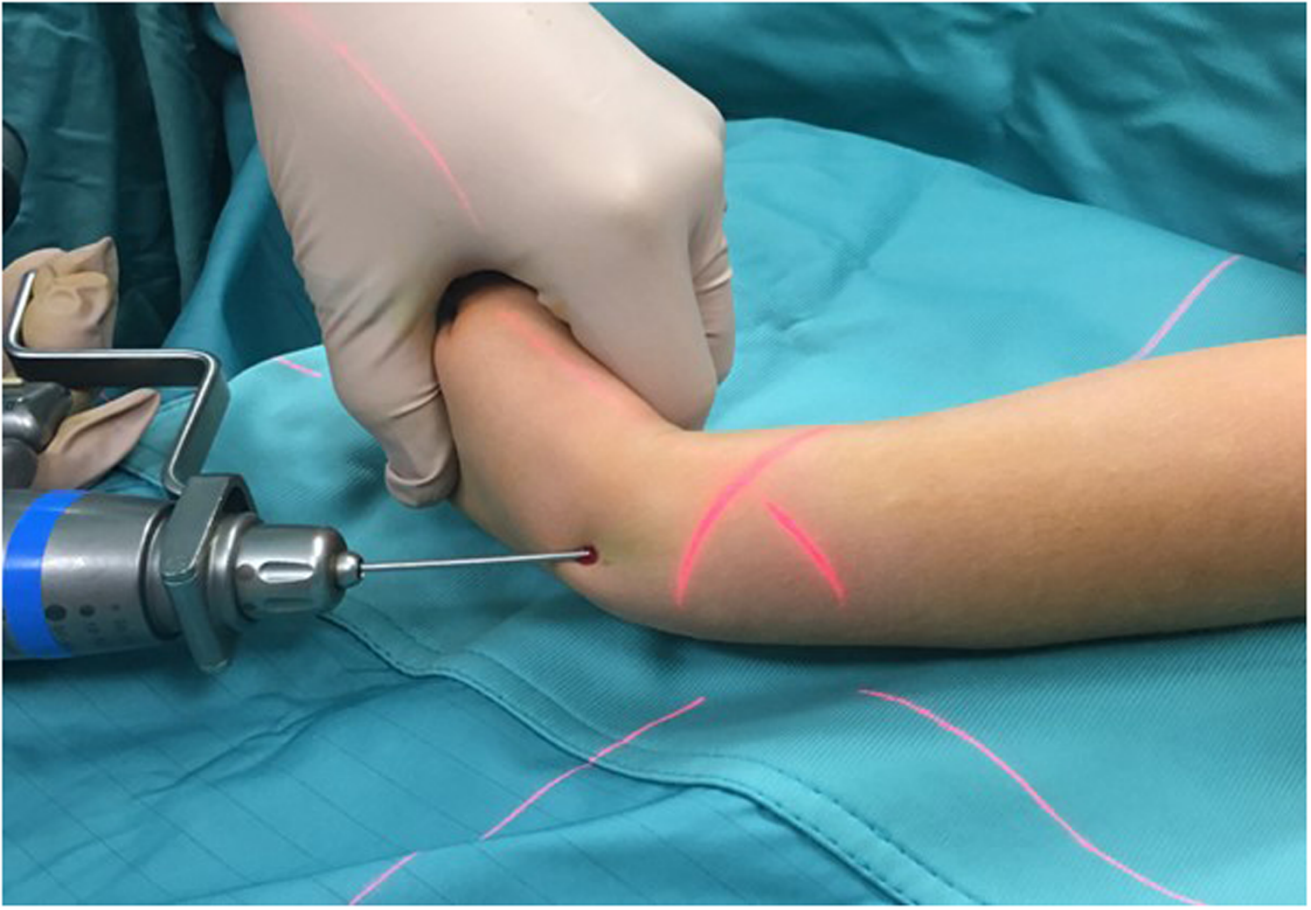
Intraoperative photograph demonstrating placement of the percutaneous pin.

**Figure 4:**
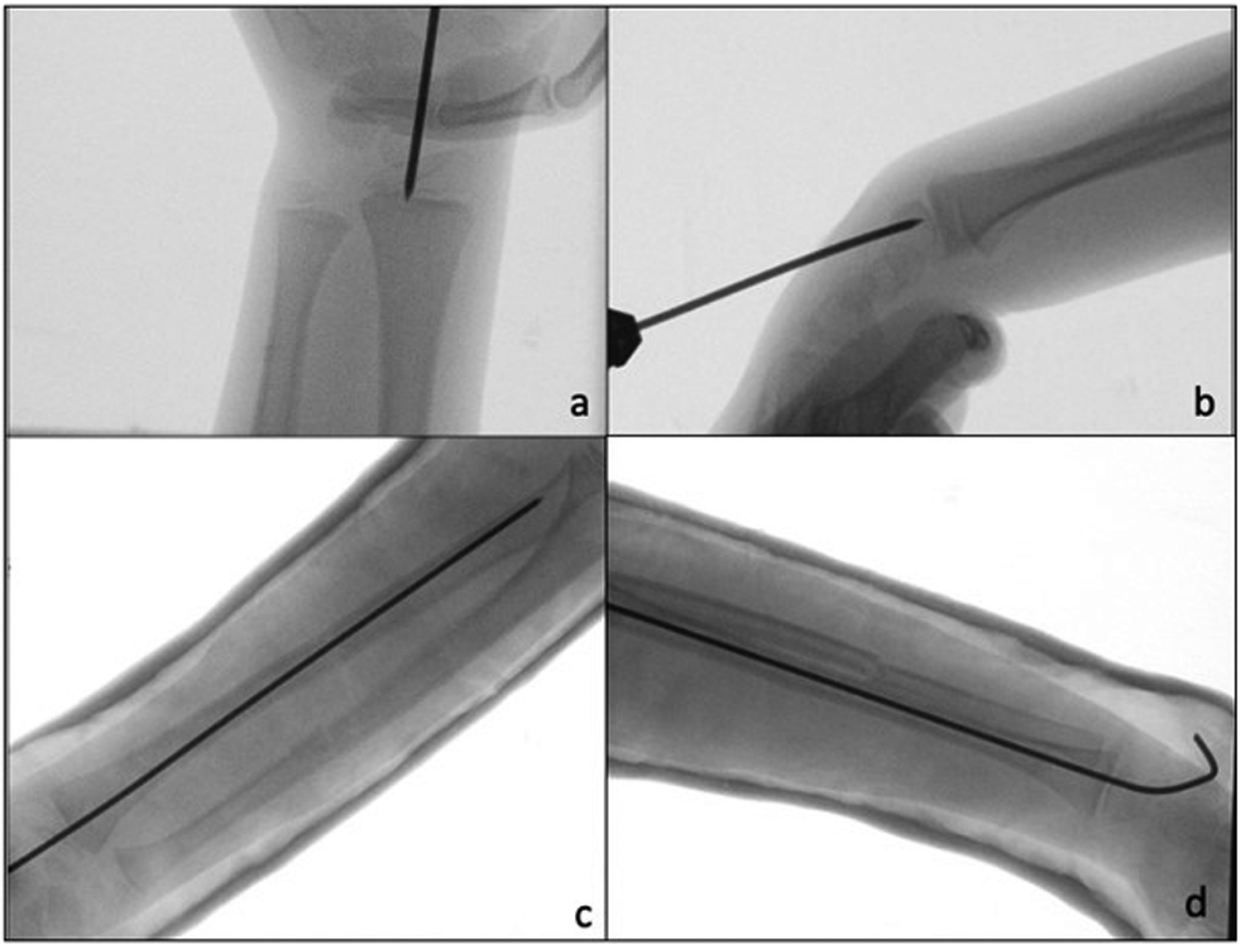
**(a,b)** Intraoperative fluoroscopic images show retrograde pinning through the radio-carpal joint. **(c,d)** Post-operative x-ray A/P and lateral, after plaster cast application.

All surgical procedures were performed under general anesthesia. A single shot dose of cefazolin (25 mg/kg) was administered intravenously 30 min before the start of the procedure. The affected limb was positioned on a radiolucent operating table. According to the width of the medullary canal and the patient's age, a K-wire (single trocar, non-threaded) with the appropriate diameter (2.0 or 2.5 mm) was used. Closed reduction was first attempted using fluoroscopy. In case of non-reducible fractures or unacceptable closed reduction, a small skin incision was made (3–4 cm), and the site of the radius fracture was exposed with minimal periosteum damage. Radial pinning was performed by retrograde access: the K-wire was applied through the radio-carpal joint, with the wrist flexed from the center and perpendicularly to the joint surface (Figures 3, 4a,b). The K-wire was inserted into and advanced along the medullary canal up to the hedge of the fracture site. Once reduction was confirmed, the K-wire was advanced across the fracture site to the opposite fragment metaphysis (Figures 4c,d). The central entry point of the K-wire is necessary for its correct and easy insertion into the canal. An eccentric entry point often leads to the creation of false paths on the opposite cortical side. In cases with both bone forearm fractures, the radius was stabilized first and the ulna only if the fracture was unstable. Ulnar ﬁxation was done in an antegrade manner through the apex of the olecranon. At the end of the procedure, the portion of the K-wire that came out from the skin was folded about 90 degrees higher than the cutaneous plane after a surgical dressing was practiced. The wrist was immobilized in a plaster cast at 30 degrees of flexion to avoid skin decubitus.

## Results

The mean duration of hospitalization was 2.5 days (range: 2–3 days). In 9 patients (35%), six affected by biosseus fractures and three by monostotic fractures, an open surgery reduction was necessary (all the fractures presented a translation of bone fragments >80% of the bone diameter). The mean duration of surgery was 25 min (range: 15–46 min), while the mean duration of anesthesia was 31 min (range: 24–73 min). The most extended procedures were those in which an open reduction was performed, and muscle tissue was found between the bone fragments. According to Daruwalla's criteria, clinical data underlined excellent results in most patients: 24 patients were excellent, two were good, and there were no poor results in the six-month follow-up. At the 6-month follow-up, two patients (7%) had a deficit of pronation and supination of less than 10 and 15 degrees. A residual radial and ulnar angulation value of less than 5 degrees was observed in 1 patient (6%). In 2 patients (7%), a radio-ulnar discrepancy of less than 6 mm was found. There was no radio-ulnar cross-union or heterotopic ossification visible at the x-ray follow-up. Radiographic healing was achieved in all cases at three months, with a mean consolidation time of 7 weeks (5.7–11 wk) (Table 1). One patient had a re-fracture about four months after K-wire removal. This patient was successfully treated with closed reduction and a further K-wire fixation. We had no cases of delayed union, growth disturbance, post-operative compartment syndrome, surgery complications, superficial or deep infection, or soft tissue irritation at the entry point of wire, elbow, or wrist stiffness. Peripheral vascular-nervous deficits were not observed.

**Table 1:** The table presents the clinical and radiographic data collected 12 months after the surgical intervention.

Post-operative complication	0
Non union or mal union	0
Pronation/Supination deficit (<15°)	2 (7%)
Residual ulnar angle (<5°)	1 (6%)
Residual radial angle (<5°)	0
Mean value in degrees	0,582 ± 1.341 SD
Forearm overgrowth	
- Maximal overgrowth 6 mm (mean value in mm)	0
- Maximal radio-ulnar discrepancy 5 mm (mean value in mm)	2 (7%)
Delayed union	0
Growth disturbance	0
Post-operative compartment syndrome	0
Surgery complications	0
Superficial infection	0
Elbow or wrist stiffness	0
Mean (range) time to union (in week)	5.9–11

### MRI evaluation

13 of 26 scans were completed without patients'sequelae. An asymmetrical bridge that did not correspond to the wire position was found in three patients. In 4 patients, no signal alterations of the physis were found; however, the affected wrist showed a worsening of the negative ulnar variance due to the fracture, unrelated to the wire position. In 1 patient, an epiphyseal cleft was found without other anomalies (such as epiphyseal deformity or abnormal signal, epiphysiodesis, or negative ulnar variance). In 1 patient, a longitudinal not ossified scar -hyperintense in 3D VIBE and T2 3D STIR, iso/hypointense in 3D DP sequences- was found involving radial distal metaphysis and epiphysis, extended to the scaphoid bone; a focal bridge at the wire position was also detected. In addition to the epiphyseal cleft, asymmetric growth of the radial epiphysis was also evident, with flattening of its ulnar aspect due to vascular damage. In 1 patient, an epiphyseal cleft without bone bridging was found. The distal metaphysis adjacent to the growth plate showed a wide linear signal alteration hyperintense in T2 3D STIR- parallel to the growth plate, resulting from vascular damage. An associated flattening of the ulnar aspect of the radial epiphysis was detected due to vascular damage. No negative ulnar variance was found. None of our patients showed wrist effusion. Table 2 illustrates the parameters evaluated in the MRI study and their corresponding values. Table 3 presents the descriptive statistical analysis of the data obtained from the MRI study*.* All correlations were calculated using Pearson's test (Table 4). The correlation between the presence of the bony bridge and the variables age, type of fracture, wire diameter, type of reduction, WTPA, and PA/KD was evaluated. The only two significant correlations identified were those between the presence of the bony bridge and the patient's age, the diameter of the Kirschner wire used and the WTPA, respectively. Considering the ratio between the wire diameter and the average surface area of the physis for age measured on axial cuts, the MRI study demonstrates that the percentage of residual cartilage lesions was less than 1% in all the cases. Furthermore, no correlation was found between the latter and the type of reduction.

**Table 2:** The table illustrates the parameters assessed in the MRI study and the corresponding values obtained.

Pt	Age	Sex	Fracture side	Fracture type	Reduction	WTPA (mm^2^)	K diameter (mm)	PA/KD Ratio	Bone bridge	Bone Edema	Epiphyseal dysmorphism	R/U discrepancy	CT (weeks)
1	9	m	Left	BB	Closed	499	2	0.629	0	0	0	0	8
2	8.5	m	Right	RO	Closed	360	2	0.872	0	0	0	0	6.9
3	9.3	m	Right	BB	Open	435	2	0.721	0	0	1	0	5.8
4	8	f	Right	RO	Closed	380	2	0.826	0	0	0	0	6,2
5	9.2	m	Left	BB	Closed	456	2	0.688	0	0	0	0	6
6	10.1	f	Right	BB	Closed	426	2	0.737	0	0	0	1	7.2
7	9.3	f	Left	BB	Closed	468	2	0.67	0	0	0	0	9.9
8	10.4	m	Right	RO	Open	501	2	0.626	0	0	0	0	6
9	13	m	Right	BB	Closed	601	2.5	0.815	1	0	0	0	7.3
10	11.3	m	Left	BB	Closed	504	2	0.623	1	0	0	1	11
11	13.1	f	Right	BB	Open	563	2.5	0.87	1	0	0	0	9.4
12	10.3	m	Right	RO	Closed	456	2	0.688	0	0	0	0	6.5
13	9.7	f	Left	BB	Open	415	2	0.756	0	0	0	0	6.7
14	8.3	m	Right	BB	Closed	380	2	0.826	0	0	0	0	6.3
15	10.1	f	Right	BB	Closed	431	2	0.728	0	1	0	0	7.2
16	9	f	Left	BB	Closed	425	2	0.738	0	0	0	0	7.5
17	8.1	m	Left	RO	Closed	392	2	0.801	0	0	0	0	8
18	9	m	Right	RO	Open	428	2	0.733	0	0	0	0	7.2
19	8.1	m	Left	RO	Closed	397	2	0.79	0	0	0	0	7
20	11.1	m	Right	BB	Open	531	2	0.591	0	0	0	0	6.5
21	10.4	f	Right	RO	Closed	501	2	0.626	0	0	0	0	7
22	8.4	m	Left	BB	Open	385	2	0.815	0	0	0	0	7,4
23	9.2	m	Left	BB	Closed	442	2	0.71	0	0	0	0	7
24	10.1	f	Right	BB	Closed	463	2	0.678	0	0	0	0	5.4
25	10.2	m	Right	BB	Open	508	2	0.618	0	0	0	0	7
26	13	m	Right	RO	Open	591	2.5	0.829	1	0	0	0	8

**Table 3:** The table presents the descriptive statistical analysis of the data obtained from the MRI study.

	Patient	Age	Fracture type	Reduction	WTPA (mm^2^)	K diameter (mm)^a^	PA/KD Ratio	Bone bridge	Bone Edema	Epiphyseal dysmorphism	R/U discrepancy	CT (weeks)
Number of values	26	26	26	26	26	26	26	26	26	26	26	26
Mean	12.77	9.854	0.3462	0.3462	459.2	2.058	0.7309	0.1538	0	0.03846	0.07692	7.246
Std. deviation	7.906	1.479	0.4852	0.4852	65.16	0.1629	0.08422	0.3679	0	0.1961	0.2717	1.264
Std. error of mean	1.55	0.2901	0.09515	0.09515	12.78	0.03195	0.01652	0.07216	0	0.03846	0.05329	0.2479

The only significant correlation was between bone bridging and the patient's age.

WTPA, wrist total physeal area; PA/KD ratio, physeal area/Kirschner diameter ratio; BB, both bones; CT, consolidation time.

^a^ 2 mm = 3.14 mm^2^; 2.5 mm = 4.90 mm^2^.

**Table 4:** The table shows the correlations calculated using Pearson's test.

	Bone bridge vs. Age	Bone bridge vs. Fracture type	Bone bridge vs. Reduction	Bone bridge vs. WTPA (mm^2^)	Bone bridge vs. K diameter (mm)	Bone bridge vs. PA/KD Ratio
Pearson *r*						
*r*	0.8073	−0.08618	0.1379	0.7047	0.847	0.2753
95% confidence interval	0.6110 to 0.9101	−0.4582 to 0.3116	−0.2635 to 0.4986	0.4365 to 0.8579	0.6841 to 0.9294	−0.1254 to 0.5988
*R* squared	0.6517	0.007427	0.01901	0.4966	0.7174	0.07581
*P* value						
*P* (two-tailed)	<0.0001	0.6755	0.5017	<0.0001	<0.0001	0.1734
*P* value summary	****	ns	ns	****	****	ns
Significant? (alpha = 0.05)	Yes	No	No	Yes	Yes	No
Number of XY Pairs	26	26	26	26	26	26

The significant correlations identified are between the presence of the bony bridge and the patient's age, the diameter of the Kirschner wire used and the WTPA, respectively.

## Discussion

The MRI study has validated the safety of the surgical technique, showing that transphyseal pinning was not associated with early or late complications affecting the distal physis of the radius. No growth plate disturbance related to the passage of the wire through the physis was detected. The study assessed the correlation between wire diameter and the percentage of lesion tolerated by the growth plate. To our knowledge, no recent studies in the literature, with a valid sample of patients, address the safety issue of transphyseal distal radius pinning with MRI evaluation.

To specifically avoid the wrist fracture healing process near the growth plate being a bias of the study, we enrolled only patients with mid-shaft forearm fractures. The surgical management of pediatric diaphyseal forearm fractures is currently controversial (21). The advantages related to the juvenile skeleton, such as remarkably rapid healing times and the minimal percentage of complications of cast immobilization (joint stiffness, loss of bone mass, functional limitations, phlebitis, Sudeck syndromes, etc.), allow for adequate compensation of traumatic deformities such as angulation and displacement and have directed almost all therapeutic choices towards the conservative treatment for many years (22, 23). The patient's age and injury site influence the fracture's healing process. The remodeling capacity depends on the proximity of the fracture to the physis: it is greater if the fracture is close to the growth plate (24). When the fracture involves the middle third of the forearm, and the patient is older than ten years, the remodeling capacity decreases, and a higher incidence of residual angulation and/or malrotation is often observed at follow-up (25, 26). Closed reduction and plaster cast immobilization are the gold standard treatments for stable and minimally displaced forearm fractures (27). This treatment is successful in most patients under 8, with no bayonet apposition or angular deformity less than 10–15 degrees (3, 28). Surgical treatment is indicated in cases of instability, open fracture, nonunion, irreducibility, severe soft tissue injury, or progressive loss of reduction in cast (27, 29). Several techniques have been proposed and practiced for diaphyseal forearm fracture treatment in children in the last two decades. Intramedullary (IM) fixation is currently the most commonly preferred method (30). The use of plates, screws, and external fixators is indicated only in limited cases (31, 32). The intramedullary osteosynthesis of pediatric forearm fractures has not been recently introduced but has become more popular in the last decade. The most widespread technique in this context is the elastic nails method (ESIN or Nancy nails) (10, 11, 33–35). This procedure respects the physis: the radius nail is implanted from the proximal metaphysis through a lateral approach (radial styloid) or a dorsal approach (Lister's tuberculum radii); it allows a limited dissection to achieve a shorter duration of anesthesia and surgery, to maintain alignment and stability of the fragments, but it requires additional anesthesia and surgery for nail removal.

The use of percutaneous pinning is an unusual method in the treatment of diaphyseal forearm fractures, whereas it is a widespread approach for treating displaced or unstable physeal or juxtaphyseal fractures in pediatric and adolescent patients. The use of temporary pinning across the physis, with smooth wires, finds consensus in the literature and is widely practiced to date (36), as is frequently done for Salter-Harris type physeal fractures (37) or Gartland II-III supracondylar humerus fractures (38, 39).

Choi et al. (1995) (40), Yung SH et al. (1998) (30), and Yung PSH et al. (2004) (41) described in their studies, respectively 157, 57, and 84 cases of pediatric forearm fractures treated by percutaneous transphyseal intramedullary Kirschner wire pinning (1.6 mm or 1.2 mm of diameter). The K-wire was introduced from the radial styloid and was pushed across the physis. Despite this, these studies have already shown, with clinical and radiographic investigations and a mean follow-up of 20 months, that transphyseal pinning was not associated with long-term outcomes affecting cartilage growth. They did not highlight nonunion, premature epiphyseal closure, or deep infection. It should be noted, however, that the potential limitation of these studies is that the follow-up was conducted only with x-rays. Şahin et al. (2017) (12) analyzed the radiographic and clinical results of pediatric diaphyseal forearm fractures treated with titanium nails or K-wires. They demonstrated no significant difference between the two procedures regarding the union time of fractures, rate of postoperative complications, range of elbow motion, and postoperative symptoms.

There is currently no consensus regarding the impact of the K-wire on physeal injury. Some studies have shown how the K-wire placed across the physis could contribute to physeal arrest (42). In the past, the relationship between transphyseal pinning and physeal growth disturbances has been a topic of investigation in several animal studies, suggesting a potential correlation between the placing of K-wire across the physis and physeal damage (13, 43). Certain factors, such as size, location, number of passes, and number and type of K-wires, may contribute to physeal injury. Other studies, however, have shown that minor central disruptions of the physis do not cause an alteration of the physeal growth (14, 44).

In the absence of reliable data regarding the passage of fixation devices through the growth plate, most surgical techniques used for treating radius and ulna fractures are based on absolute respect for the growth cartilage.

Magnetic resonance imaging is currently considered the most suitable examination for studying cartilage tissue. MRI allows an accurate, radiation-free, volumetric evaluation of the physis.

Shi DP et al., in 2009, showed that MRI accurately documents defects of physis more clearly than other exams in physeal injury (16). Jaramillo et al. described the utility of MRI in evaluating physeal injuries. They reported that MRI could be functional in minor disruptions of the growth cartilage detection and can show early cartilaginous and vascular changes that lead to growth disturbances (17).

Our MRI exam protocol included 3D Volumetric Interpolated Breath-hold Examination (VIBE), 3D Proton density (DP), and 3D T2 SPACE Short Tau Inversion Recovery (STIR), with a total exam time between 10 and 15 min. VIBE sequence is a form of volumetric imaging using fast 3D gradient-echo (GRE) sequences that produce T1 weighted images (TR 5.17 msec – TE 2.2 msec). This high intrinsic contrast resolution sequence allows differentiation between cartilage (hyperintense) and bone or bony bridges (hypointense areas). Proton density sequences provide (TR 1.100 msec, TE 41 msec) excellent bone, periosteum, and cartilage anatomic details. T2 3d STIR (TR 2.900 msec, TE 203 msec – inversion time 160 msec) is a high contrast fluid-sensitive sequence that best detects bone marrow edema. High-resolution 3D comparative MRI is essential in evaluating the radial growth plate due to its oblique orientation and regular undulation that increases with age in response to physiologic dynamic biomechanical forces.

Moreover, 3D MRI imaging can provide quantitative data about the physis and can differentiate a physiologic bridge, due to its imminent closure, from an iatrogenic bridge caused by the K-wire. T1-weighted sequences are more helpful in distinguishing the open and closed parts of the physis (45). The disadvantages of MRI are its expense, limited availability, and the necessity for sedation of younger children (for this reason, patients under six were excluded from the study).

From the descriptive statistical analysis of our study, it emerged that the significant correlations found are between the presence of bone bridge and the variables patient's age, diameter of the Kirschner wire used, and the WTPA. However, given that in all cases, the location of the bony bridge does not correspond to that of the Kirschner wire passage, the most realistic interpretation is that this alteration corresponds to the initial phase of the growth plate closure process. In a similar way, the correlation between the presence of the bone bridge and the WTPA (wrist total physeal area) may depend on the older age of patients with a wider WTPA.

Therefore, about what has just been said, considering the diameter of the K-wire used (2/2.5 mm) and that our MRI study has highlighted that the percentage of residual cartilage lesions was less than 1% in all the cases, this technique should be considered safe.

**Figure 5:**
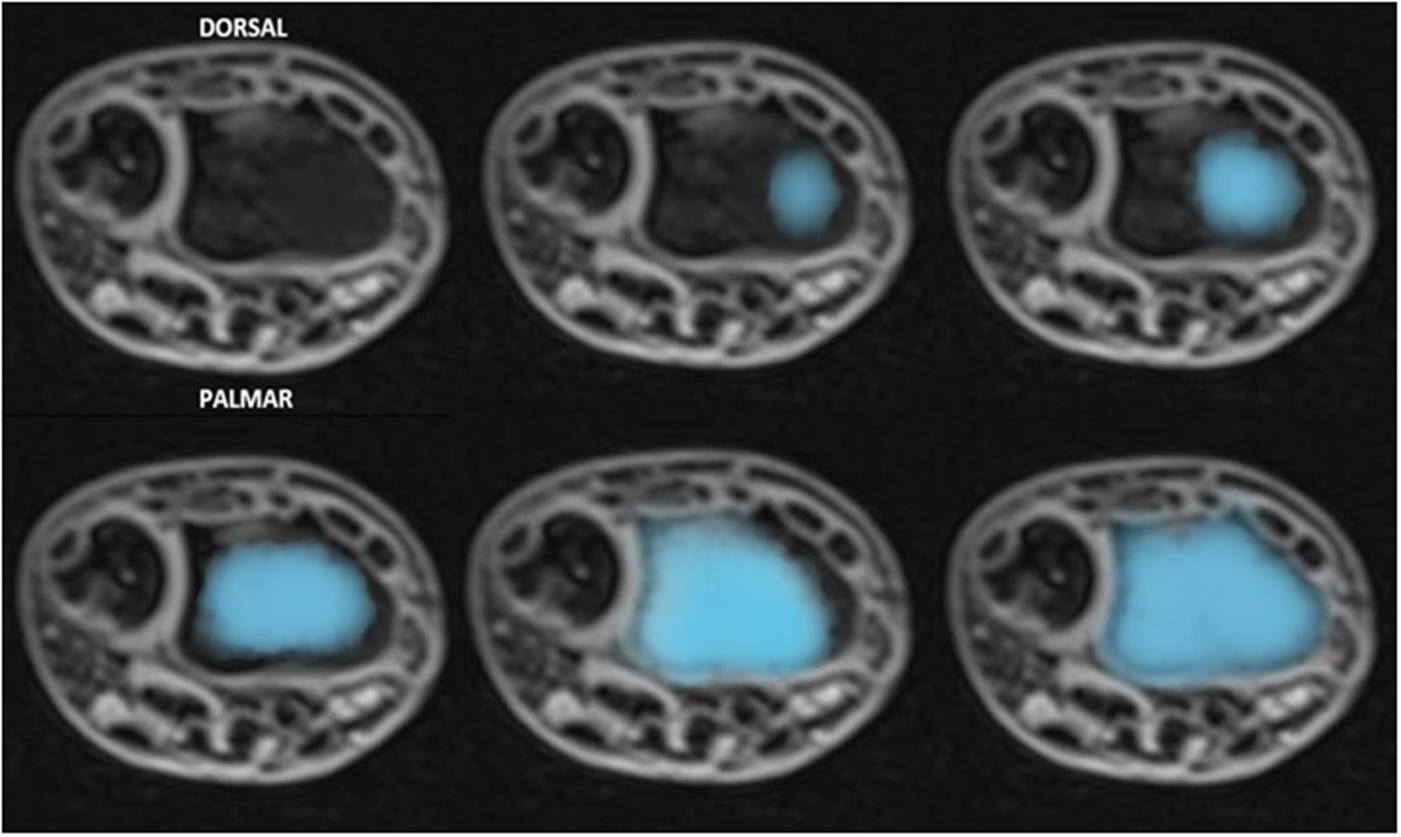
The figure shows a reproduction of the sequential assessments of dynamic physeal closure of the distal radius illustrated by kraus et all: it begins centro-radial and ends with a radio-dorsal bridge.

Another aspect to consider is the physiological closure of the distal growth plate of the radius. Kraus et al. (45) described in their work that the starting point of the closure of the growth plate could coincide with the area with the least blood supply of the physis. They evaluated the dynamic of the distal radius's physeal closure, and the MRI sequential assessments showed that bony bridging of the distal radius physeal plate begins centro-radial and ends with a radio-dorsal bridge (Figure 5).

Therefore, the entry point of the Kirschner wire, in the central part of the epiphysis, should reduce the risk of damage to the growth plate. The central entry point of the K-wire is necessary for its correct and easy insertion into the canal. An eccentric entry point often leads to the creation of false paths. Furthermore, the direction of the wire should be perpendicular to the growth plate and placed in the center of the medullary cavity. In this way, using a smooth fixation device, the growth plate retains the ability to grow physiologically, minimizing the possibility of growth disturbances. On the contrary, the growth process might be altered when the K-wire is fixed obliquely in the cortical bone. Moreover, the oblique orientation would involve a larger portion of the physis.

Lastly, another aspect worth considering is the shape of the radio-carpal joint, which consists of two independent articular surfaces, the scaphoid facet (laterally) and the lunate facet (medially). Because of that, the K-wire central entry point allows any possible damage to only hit a small peripheral portion of one or the other. Moreover, premature epiphyseal closure is rare despite the frequency of physeal fracture of the distal radius, unlike distal femoral epiphyseal separations, which are less common but have a high rate of complication (46).

According to Dorman et al., the K-wire fixation did not increase the risk of growth arrest (15).

In their 2008 study, Smith et al. (47) analyzed five cases of pediatric unstable upper extremity fractures were treated with transphyseal pinning, which included two Gartland type III supracondylar humerus fractures, one Gartland type II supracondylar humerus fractures fracture, one both bone forearm fracture, one distal radius physeal fractures. They evaluated the physis using MRI at three and six months after the removal of the K-wire, identifying only one case of physeal bridging that was believed to be related to the trauma (a Salter-Harris type II fracture of the distal radius). Therefore, this study, despite being limited by the small sample of patients, has shown that temporary pinning across the physis did not necessarily cause physeal damage.

Our study has limitations, including the retrospective nature and a relatively small number of patients. However, the study results confirmed that transphyseal K-wire could be safely used, and it is a valid and minimally invasive surgical technique. In some cases, transphyseal intramedullary fixation could be an alternative surgical treatment for forearm shaft diaphyseal and distal radius fractures. It is easy to perform, has several advantages, and has a low complication rate. Furthermore, the percutaneous technique does not require the skin incision for surgical exposure and additional surgery and/or anesthesia for wire removal.

## Conclusions

The MRI study has validated the safety of this surgical technique and confirmed that the distal radius growth plate better tolerates injury compared with other physis. It has demonstrated the absence of long-term damage to the cartilage. It confirms, as other studies, that the growth plate could be violated if some technical and biological criteria are respected:

− To use a non-threaded K-wire;
− To consider the ratio between K-wire diameter and average surface area of the physis according to the age (2 mm/2.5 mm);
− No more than one passage through the growth plate to avoid cartilage damage;
− To enter the central part of the radial epiphysis in both radiographic views;
− The direction of the wire has to be perpendicular to the growth plate.

This study demonstrates that the percutaneous trans-physeal technique could become a valid alternative to the standard method, offering a rapid learning curve, shorter surgical times compared to ESIN, and reduced healthcare costs. The removal of the wire does not necessitate a skin incision or additional interventions and anesthesia. Given that most surgical techniques for treating radius and ulna fractures emphasize strict adherence to the integrity of the growth cartilage, this study may pave the way for the broader adoption of transphyseal Kirschner wires in the treatment of specific upper limb fractures (such as those involving the wrist and forearm), challenging the longstanding dogma surrounding the inviolability of growth cartilage in pediatric patients.

## Data Availability

The raw data supporting the conclusions of this article will be made available by the authors, without undue reservation.
